# Supplementary material for: Endoscopic papillary balloon dilatation less than three minutes for biliary stone removal increases the risk of post-ERCP pancreatitis
Source: PLoS One. 2020 May 26;15(5):e0233388. doi: 10.1371/journal.pone.0233388 (PMC7250446; doi:10.1371/journal.pone.0233388)
Supplement: Attachment — Submitted filename: Respone to Reviewers.docx [file pone.0233388.s001.docx]

Reviewer #1:

1. Can they give information on size of balloons used for papillary dilation (what was size in large balloon dilation).

**Reply:** Thanks for your comments. We added the information in the revised table2. EPLBD was defined as balloon size more than 10mm (revised table 2).

2. Grammatical correction "systematic" instead of "systemic" in the discussion, 8th line

**Reply:** Thanks for your kind comment. We revised the grammatical error as your suggestion. (page 12, 1st line)

3. Information on complete stone removal on first attempt or repeat ERCP's were needed.

**Reply:** Thanks for your comments. Actually, all stones were removed successfully on first attempt and no repeat ERCP was done during the follow up period. The information was added in the revised table 2.

Reviewer #2:

Kuei-Chuan Lee et al. present in their manuscript “Endoscopic Papillary Balloon Dilatation less than three minutes for biliary Stone Removal Increases the Risk of Post-ERCP-Pancreatitis” interesting data from a retrospective single centre observational study on 198 patients (11/2010-10/2012). The main finding was that in patients that underwent Endoscopic Papillary Balloon Dilatation (EPBD) for stone removal, dilatation duration < 3 minutes, lower CBD diameter and younger age were in a multivariate analysis independently associated with Post-ERCP-Pancreatitis (PEP).

As the authors have mentioned in their manuscript, a number of previous studies have already shown that the duration of EPBD was inversely associated with PEP; therefore, clinical guidelines already suggest an EPBD duration of at least 2 minutes.

The authors furthermore emphasize that no studies investigated so far, the impact of large balloon dilatation (EPLBD) on PEP. Nevertheless, the findings presented here are indeed not surprising but rather confirm previous studies.

It is unclear to me, why the authors choose the time intervals at 3min and 5min dilatation time to build the three groups that they compared? Why not 2min or 4 min? Was there an ROC-analysis performed to identify the optimal discriminatory time point?

**Reply:** Thanks for your comments. The time point was not set by ROC analysis. However, a randomized trial by Liao et al. found that the risk of PEP was lower with a 5min EPBD as compared with those with a 1min EPBD (Gastrointest Endosc. 2010;72(6):1154-62). Therefore, we firstly chose 5min as a discriminatory point. Then we found that the mean of dilatation duration was 208.26±4.54 seconds, and the median was 210 seconds among the patients who received balloon dilatation duration less than 5 minutes (n= 127). For the convenience in daily practical use, we then chose 3min as another discriminatory point, which near the mean or median value in the patients receiving balloon dilatation time less than 5min. Finally, the time intervals at 3min and 5min were chosen.

I also wonder why only patients between 2010 and 2012 were included. For the endpoint PEP a long follow-up is not needed and therefore there is no obvoious reason not to include also patients from the years 2013-2018. This would also allow to include protective stenting and NSAIDs as protective factors.

**Reply:** Thanks for your comments. We totally agree that protective stenting and rectal NSAIDs are important protective factors for PEP nowadays. However, during the period (2014-2016) when we retrospectively started to collect the data of patients receiving EPBD in our hospital, the use of rectal NSAIDs and stenting were still debatable. Therefore, we only chose the patients between 2010 and 2012 when no rectal NSAIDs or stenting was used in our ERCP room. Actually, we are interested in whether dilatation time could further affect the occurrence of PEP in another time period (2013-2018) when rectal NSAIDs was frequently used in high risk PEP patients in our hospital, future study will be initiated. (page 14, line 18)

Furthermore, in the Methods it is not mentioned which balloons and which sizes of balloons were used for EPBD and EPLBD. This essential information must be given.

Balloon Diameter (if different balloons were used) and also mechanical lithotripsy should additionally be included in the analysis of risk factors (table 4).

**Reply:** Thanks for your important suggestions. We had added information of balloons and size in the revised method (page 7, line 12) and Table 2. Mechanical lithotripsy had no significantly predictive value in univariate analysis (revised Table 4).

Minor limitations are the retrospective study design with a possible BIAS since the time of EPBD was not standardized but was the choice of the endoscopist.

It is also not current standard anymore not to use a guidewire for primary cannulation. However, the relatively low rate of PEP reflect the skills and experience of the endoscopist performing biliary cannulation without using a guidewire.

**Reply:** Thanks for your comments. We added these comments as study limitations in the revised discussion (page 14, line 14).

In Table 2 percentages should also be given for EPLBD.

**Reply:** Thanks for your comment, we had revised that in the Table 2 as your suggestion.

An interesting but not significant finding was that no biliary tract infection was found in group A but 4.1% in group B and 8.5% in group C. If Group A would be compared with Group B and C together was the difference than statistically significant?

**Reply:** We had analyzed that as your suggestion. However, there was no difference in biliary tract infection when compared the Group A (n=0) with the Groups B and C together (n=10) (p=0.170). (page 10, line 15).
